# Conformational Analysis of Methoxy- and Methylthio-Substituted Thiophenes by Broadband Rotational Spectroscopy and Computational Chemistry

**DOI:** 10.1021/acs.jpca.6c04394

**Published:** 2026-10-08

**Authors:** Timothy J. Barnum, Nathan Riordon, Lukas Baker, Nicholas J. Gestring

**Affiliations:** Department of Chemistry, 7254 Union College, Schenectady, New York 12308, United States

## Abstract

Functionalized aromatic heterocycles exhibit strong conformational preferences dependent on the identity of the heteroatom, substituent functional group, and substitution position. In this study, we investigated the conformational landscapes of 2- and 3-substituted methoxy- and methylthio- thiophenes by chirped-pulse Fourier transform microwave spectroscopy. One lowest energy conformation for each molecule was identified due to efficient conformational cooling in the pulsed jet. For three of the four species, the conformational preference orients the substituent group *syn* to a double bond with all heavy atoms in the plane, while 2-(methylthio)­thiophene uniquely adopts a *skew* conformation in which the barrier to methyl internal rotation is substantially reduced. Quantum chemical calculations, including natural bond orbital analysis, reveal that a balance of steric and stereoelectronic effects determines the lowest energy conformations of these species.

## Graphical abstract

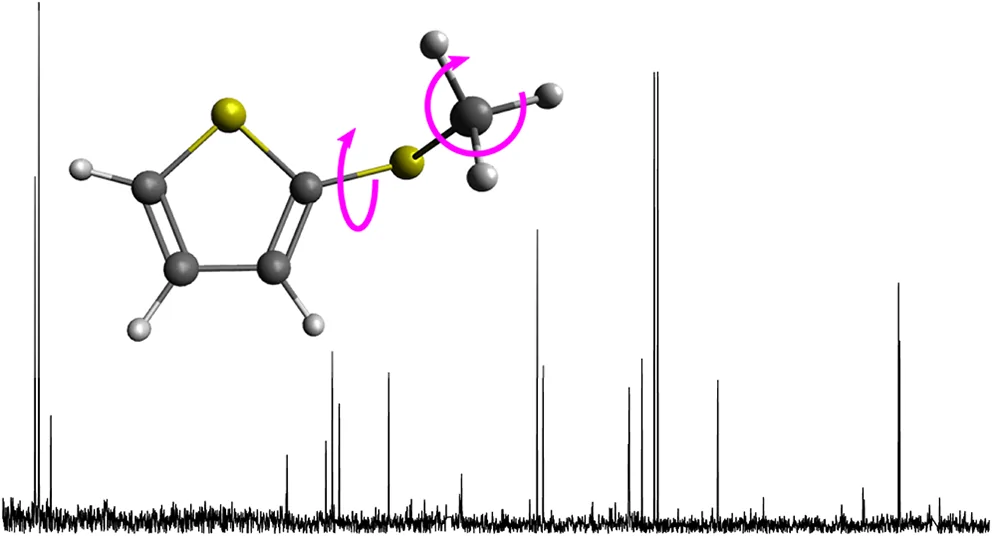

## Introduction

Heterocycles play an important role in biological molecules,^1^ pharmaceuticals,^2^ functional molecules^3–5^ and advanced materials.^6–9^ In many cases, heterocycles act as linking groups with substitution at the various positions in the ring. The identity of the heteroatom(s), the identity of the substituted functional group, and the substitution position all play key roles in dictating not just the chemical linkage, but also the chemical shape. In one example, changing imidazole to thiazole in a potent agonist for the human complement C3a receptor resulted in inhibition of the receptor, due to a conformational change at the heterocycle carboxamide linkage.^10,11^ To take advantage of the conformational directing effects of substituted heterocycles, a framework for understanding and predicting the interactions between substituent group and heterocycle is necessary. To that end, this study examines a series of methoxy- and methylthio-substituted thiophenes as model compounds. Thiophenes are themselves important heterocyclic building blocks in a wide range of applications,^12,13^ while methoxy- and methylthio-substituted thiophenes, in particular, have found applications in the production of regioregular polymers,^14^ water-soluble metal-effect paints,^15,16^ antitumor agents,^17^ and organic electronics.^18–20^

The directing effects in heterocyclic ethers have been examined computationally for a wide range of species.^21–24^ Heterocycles with a methoxy substituent *ortho* to an O or S atom favor a conformation *syn* to the C=C bond in which the C–O or C–S bond is coplanar with the C=C bond, while those *ortho* to an N atom favor the corresponding *anti* conformation. This preference has been verified by just two gas-phase high-resolution studies on 2-methoxyfuran and 2-methoxypyridine.^25,26^ The difference in heteroatom behavior has been ascribed to the relative strength of the lone pair repulsion between the ether and the heteroatom.^22^ A more detailed study identified delocalization of the ether O lone pair into the ring as the driving force for adopting a planar heavy atom structure and a more influential factor than sterics in dictating the specific *anti* or *syn* preference of a molecule.^24^ The focus of these previous studies has been on substitution at the *ortho* position, and the electrostatic, orbital, and steric impacts on conformational preferences is only partially addressed. Moreover, a dearth of information is available into the conformational state of heterocycles with thioether substituents.

Here, we investigated the conformational states of 2-methoxythiophene (2-MOT), 3-methoxythiophene (3-MOT), 2-(methylthio)­thiophene (2-MST), and 3-(methylthio)­thiophene (3-MST) by chirped-pulse Fourier transform microwave (CP-FTMW) spectroscopy and quantum chemical calculations. The structures of 2-MOT and 3-MOT have been previously examined by single crystal X-ray diffraction, in which both species were determined to adopt a conformation where the C–O bond of the methoxy group lies coplanar with the thiophene ring and oriented *syn* to the C=C double bond.^27^ In addition, ultraviolet photoelectron and electron transmission spectroscopy of 3-MOT confirmed a single conformation consistent with the *syn* geometry at room temperature in the gas phase.^28^ The same techniques applied to 3-MST identified two conformations believed to be a *syn* and a *skew* structure, in which the methylthio subsituent is oriented out of the plane of the thiophene ring, in a ratio of 60:40 *syn*:*skew*. This result is similar to a 70:30 *syn*:*skew* ratio determined in a benzene solution of 3-MST at 30 °C by dipole moment measurements.^29^ Our quantum chemical calculations predicted specific conformational preferences for 2-MOT, 3-MOT, 2-MST, and 3-MST, and were used to assign a single conformer of each species under the jet-cooled conditions of our experiment. Calculated geometries of the identified conformations demonstrate structural changes induced by substitution at the 2- and 3- positions of the thiophene ring. Additionally, for 2-MST, splitting due to internal methyl rotation was observed and fit to derive an experimental *V*_3_ barrier. Natural bond orbital (NBO) analysis provided insight into the stereoelectronic origins of the observed conformational preferences for all molecules.

## Experimental Section

A 6–18 GHz CP-FTMW spectrometer was recently constructed at Union College and is described in detail here. Samples were introduced by placing a saturated cotton ball, warmed to ∼80 °C, in line with a General Valve Series 9 pulsed valve and backed by 40 psi of argon. Gas pulses at a 20 Hz repetition rate were injected into an approximately 40 L spherical vacuum chamber which was evacuated by a magnetically levitated turbomolecular pump (Osaka TGBMW 2400), backed by a multistage roots pump (Edwards, nX90i). During operation, the chamber pressure rises to 5 × 10^–5^ Torr. The jet expansion cools the rotational degrees of freedom to an approximate temperature of 3 K.

A microwave circuit schematic of the CP-FTMW spectrometer appears in Figure 1 and a complete part list is included in the Supporting Information. Microwave pulses with a linear frequency chirp from 0.010 to 400 MHz and a duration of 10 μs were generated by an arbitrary waveform generator (AWG) and subsequently mixed in a double-balanced mixer with the fixed frequency output of a synthesized signal generator, serving as the local oscillator (LO). This up-converted pulse was then amplified in a solid-state amplifier and broadcast by a quad ridged, dual-polarization horn antenna inside the vacuum chamber. Before entering the chamber, the chirp passed through a single-pole single-throw (SPST) microwave PIN switch that blocks noise from the high power amplifier following the chirped excitation; an attenuator reduced the power output from the amplifier to a range compatible with the power handling limits of this switch. After interacting with the sample, the high power chirp was blocked from reaching the sensitive detection electronics by a diode limiter and a second switch. Approximately 300 ns after the chirp, the second switch closed and free induction decay (FID) from the molecular sample was allowed to pass through a low-noise amplifier, before down-conversion by mixing with the same fixed frequency from the synthesized signal generator. This low frequency time domain signal was directly digitized by a 12-bit 3.2 GHz digitizer. After 7812 averages of a 20 μs signal, the data was transferred to a personal computer, where multiple such acquisitions were averaged together in postprocessing. To acquire a spectrum across the full 6–18 GHz band, the LO frequency was stepped in increments of 400 MHz. Since the chirped excitation pulses span a range of 400 MHz on either side of the LO, a single acquisition is unable to distinguish frequencies observed on the high or low frequency side of the LO. By stepping the LO in an increment equal to the frequency width of one sideband, the frequency of every observed signal can be unambiguously determined. To speed up data acquisition, 14 excitation and detection cycles were repeated within every ∼800 μs gas pulse. More than one million acquisitions were averaged at each LO setting. A common timebase for the experiment was provided by phase locking the AWG and digitizer to the 10 MHz output of a rubidium frequency standard. The frequency accuracy of these measurements was estimated at 15 kHz.

**Figure 1:**
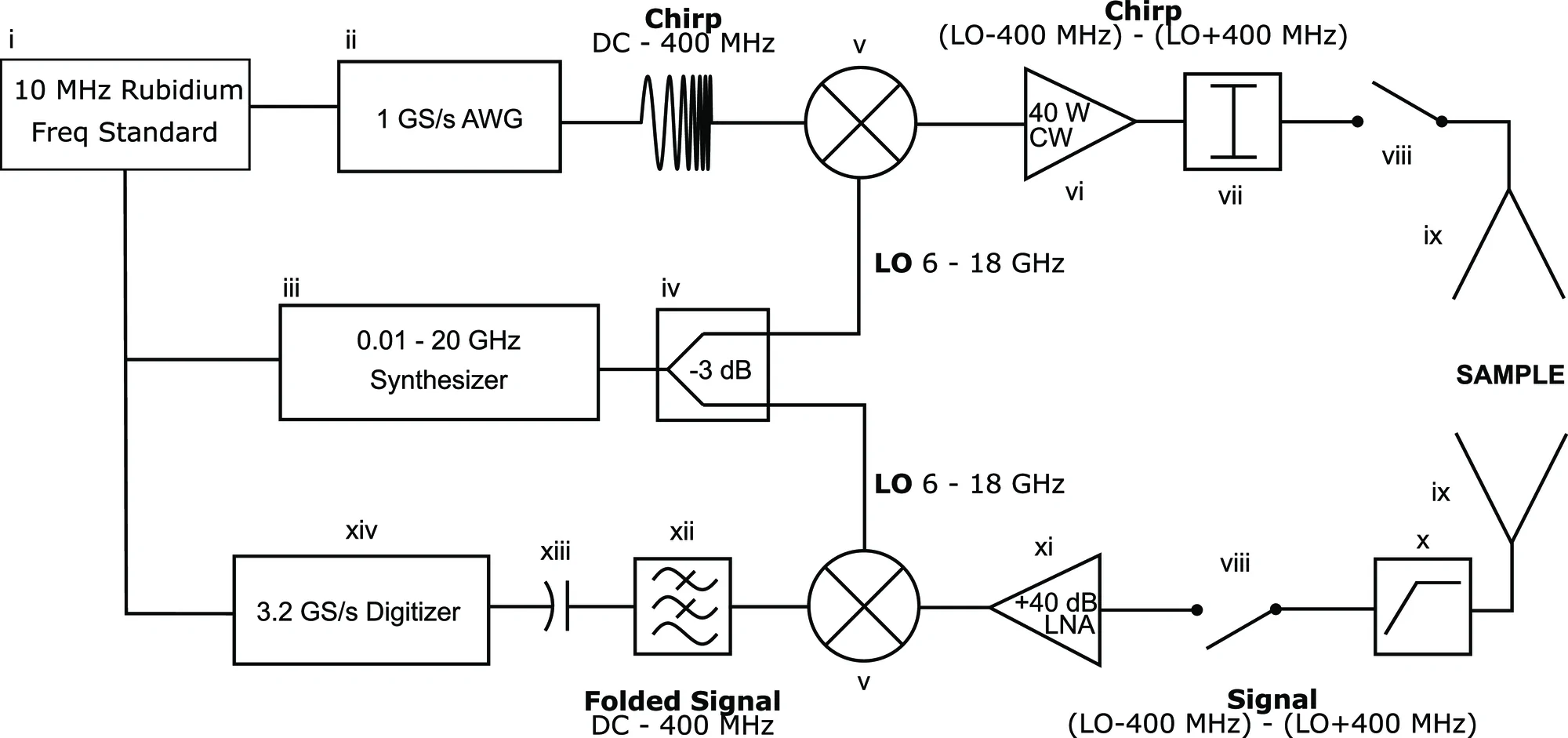
Schematic diagram of the 6–18 GHz CP-FTMW spectrometer. A 10 MHz Rb frequency standard (i) provides the time base to which the AWG (ii) and frequency synthesizer (iii) are phase-locked. The single frequency output of the frequency synthesizer (iii) is divided (iv) and mixed with the chirped pulse from the AWG (ii) in a double-balanced mixer (v) to obtain an excitation pulse in the 6–18 GHz range. This excitation pulse is amplified (vi), attenuated (vii), and passed first through a SPST switch (viii) before entering the sample chamber via a microwave horn antenna (ix). The molecular FID is detected by an identical horn antenna (ix), passed through a diode power limiter (x) and a second SPST switch (viii), and amplified (xi). The FID in the 6–18 GHz region is down-converted in a second double-balanced mixer (v) with the same synthesizer output used in the up-conversion of the excitation pulse. The low frequency signal then passes through a low-pass filter (xii) and DC block (xiii) before being directly digitized (xiv) and transferred to a PC for postprocessing. A complete part list for the spectrometer may be found in the Supporting Information.

An initial configuration in which the time base was set by the 10 MHz TCXO signal of the Valon signal generator, an approach previously described in ref 30., did not yield sufficient frequency accuracy. An offset of approximately 30 kHz was consistently observed; no attempts were made to correct for or recalibrate this 10 MHz signal.

For all experiments, samples of 2-MOT (Millipore-Sigma), 3-MOT (Millipore-Sigma), 2-MST (AA Blocks), and 3-MST (AA Blocks) were used without further purification.

For 2-MOT, additional spectroscopy was performed with a coaxial jet-cooled 75–100 GHz CP-FTmmW instrument at MIT described in detail in ref 31. Sample introduction was accomplished by placing glass wool soaked with the liquid sample in the gas line behind the pulsed nozzle, and approximately one million acquisitions were averaged.

## Quantum Chemical Calculations

To assist in the assignment and interpretation of experimental results, quantum chemical calculations were undertaken for the four molecules of interest. The potential energy landscape of each molecule was explored at the M06–2X/def2-QZVP level of theory^32,33^ by varying the dihedral angles of the two rotatable bonds corresponding to the methoxy/methylthio and methyl torsional motions. The M06–2X functional was chosen for its good performance in benchmarking studies of both thermochemistry^32,34,35^ and molecular structure^36^ for main group elements. Minimum energy pathways for the methoxy/methylthio torsion of MOT and MST are shown in Figures 2 and 3, respectively, while methyl torsion potentials are shown in Figures S1 and S2 in the Supporting Information. Minima identified on these surfaces were optimized at the same level of theory with VeryTightSCF and TightOpt settings and confirmed to be minima by harmonic frequency analysis. VPT2 calculations were subsequently performed to obtain vibrational corrections to the rotational constants and quartic centrifugal distortion constants. Due to the sensitivity of the M06–2X functional to the integration grid, the densest prebuilt grid (DefGrid3) was used for all calculations. All calculations were performed in ORCA 5.0.5 ^37^ with the exception of VPT2 calculations performed in ORCA 6.1.1.^38^

**Figure 2:**
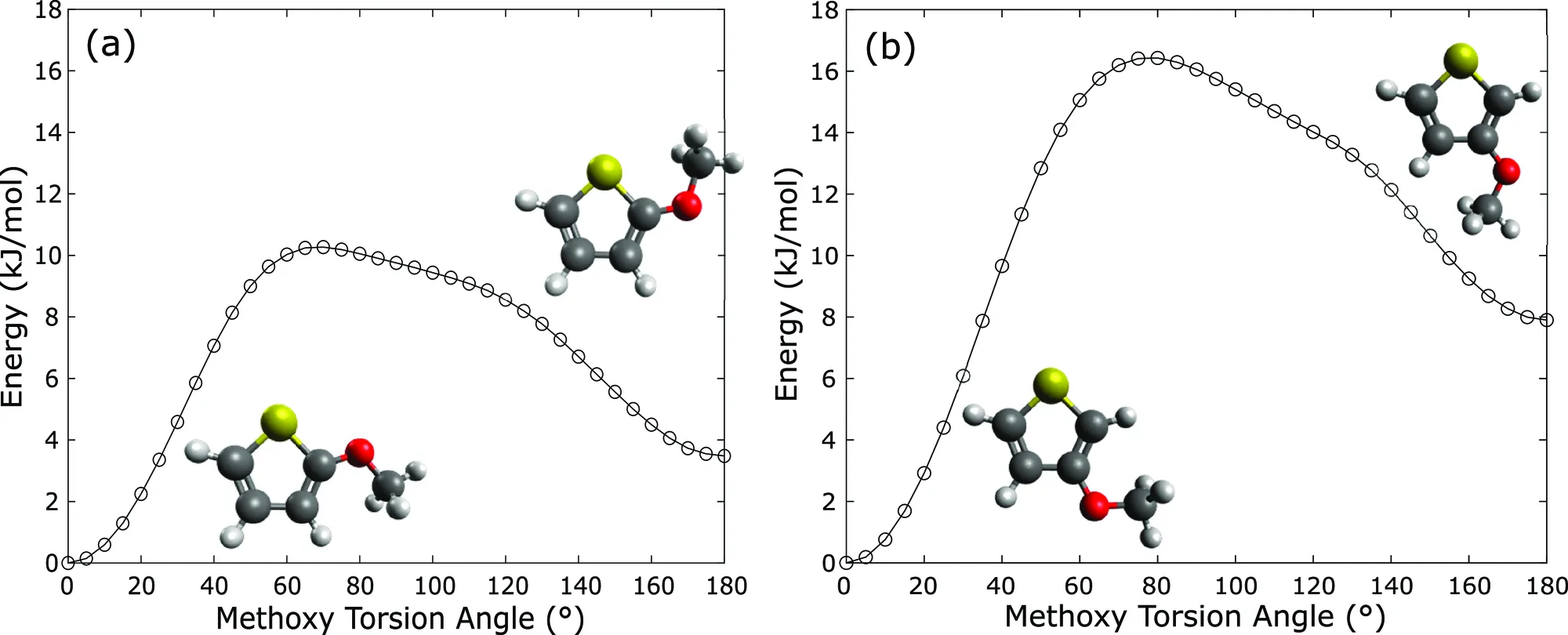
M06–2X/def2-QZVP potential energy surface along the methoxy torsion angle for (a) 2-MOT and (b) 3-MOT. In both species, the conformation *syn* to the double bond is lower in energy.

**Figure 3:**
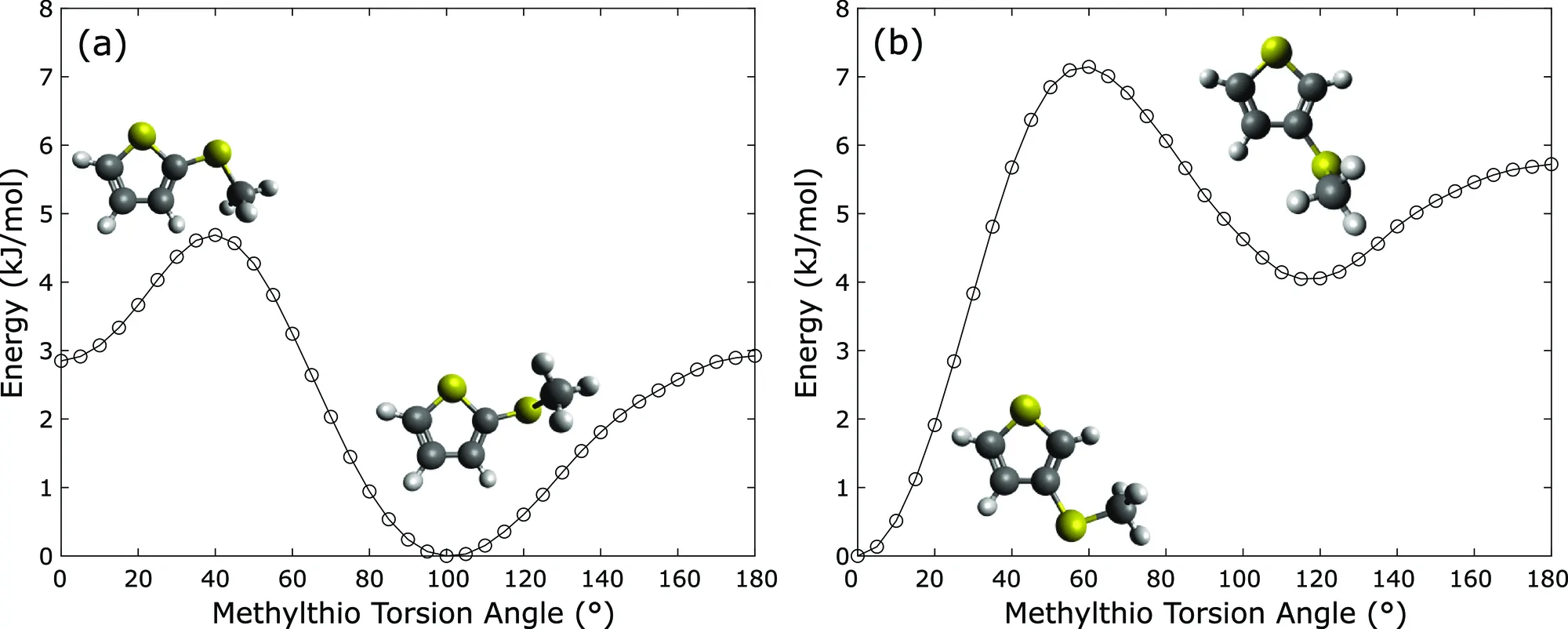
M06–2X/def2-QZVP potential energy surface along the methylthio torsion angle for (a) 2-MST and (b) 3-MST. A new *skew* conformation is preferred for the 2-substituted species, while *syn* to the double bond remains lowest in energy for the 3-substituted species.

The potential energy surface in Figure 2 identifies two conformations each for 2-MOT and 3-MOT in which the C–O bond of the methoxy group lies coplanar with the thiophene ring. For both molecules, the methoxy group is oriented *syn* to the C=C bond in the lowest energy conformation, and modest barriers of less than 10 kJ/mol exist for interconversion from the higher to lower energy conformation. The zero point energy (ZPE) corrected energy difference between *syn*- and *anti*-2-MOT is just 2.8 kJ/mol. Surprisingly, the 3-MOT exhibits a much stronger conformational preference with a ZPE corrected energy difference of 7.5 kJ/mol between conformers. Based on these calculations, the *syn* conformation of both species is expected to dominate under the cold (∼3 K) conditions of the gas expansion.

Replacing the methoxy substituent with a methylthio group results in distinctly different conformational preferences. As shown in Figure 3, the results of a relaxed scan along the methylthio torsion angle reveal a new minimum for both species in an out-of-plane *skew* orientation, while the corresponding higher energy *anti* conformer in MOT becomes a saddle point on the potential energy curve in MST. The calculated geometries of these two new minima are similar with a methylthio torsion angle of approximately 99° and 115° above the plane relative to the C = C bond for 2-MST and 3-MST, respectively. In 2-MST, this new *skew* geometry is predicted to be the lowest energy conformation by 3.6 kJ/mol. The *skew* and *syn* conformations of 3-MST have a similar ZPE corrected energy difference of 3.3 kJ/mol, but the *syn*-3-MST is predicted to be the more stable species at this level of theory. These results for 3-MST are in contrast with a previously reported potential energy curve at the MP2/6–31G­(d,p) level of theory where the *skew* geometry was predicted to be lower in energy by about 3 kJ/mol.^28^ Interestingly, the MOT potential energy curves exhibit a shoulder just above 100° along the torsional coordinate in the approximate location of the new *skew* geometry in MST. The energetic factors leading to these unique conformational preferences are discussed below.

As shown in Figures S1 and S2, the barriers to methyl internal rotation for all of the species with a heavy atom planar structure are greater than 10 kJ/mol, and thus not expected to influence the rotational spectra at the resolution of our experiment. In contrast, the *skew* conformations of 2-MST and 3-MST exhibit substantially lower barriers. A fit to eq 1 predicts barriers of 6.69 and 6.47 kJ/mol for 2-MST and 3-MST, respectively. The spectra of the *skew* conformations of these molecules are expected to contain resolvable splittings due to internal rotation.

1 $$V\left(\alpha \right)=\frac{V_{3}}{2}\left(1-\cos3\alpha \right)$$

## Results and Discussion

### Spectral Analysis

The raw FID data were Fourier transformed over a 20 μs window with a Hann-type windowing function to suppress side lobes, leading to line widths of approximately 100 kHz full width at half-maximum (fwhm) at 10 GHz. A full spectrum across the 6–18 GHz range was stitched together by harmonic averaging of the frequency domain spectra at each LO setting. The full 6–18 GHz spectrum for 2-MST is shown as an example in Figure 4a. Peaks in the experimental spectra were fit to a Gaussian line shape to extract the center frequencies. Rigid rotor fits to a Watson *A*-reduced, *I* ^*r*^ representation Hamiltonian were performed using the PGOPHER software package.^39^ Rigid rotor fits in the *S* reduction yielded similar quality fits and are shown in Tables S1, S2, and S3 in the Supporting Information. A minimal set of quartic centrifugal distortion constants was included to obtain a fit error consistent with the estimated line accuracy, while the unfit constants were fixed to the values from quantum chemical calculations.

**Figure 4:**
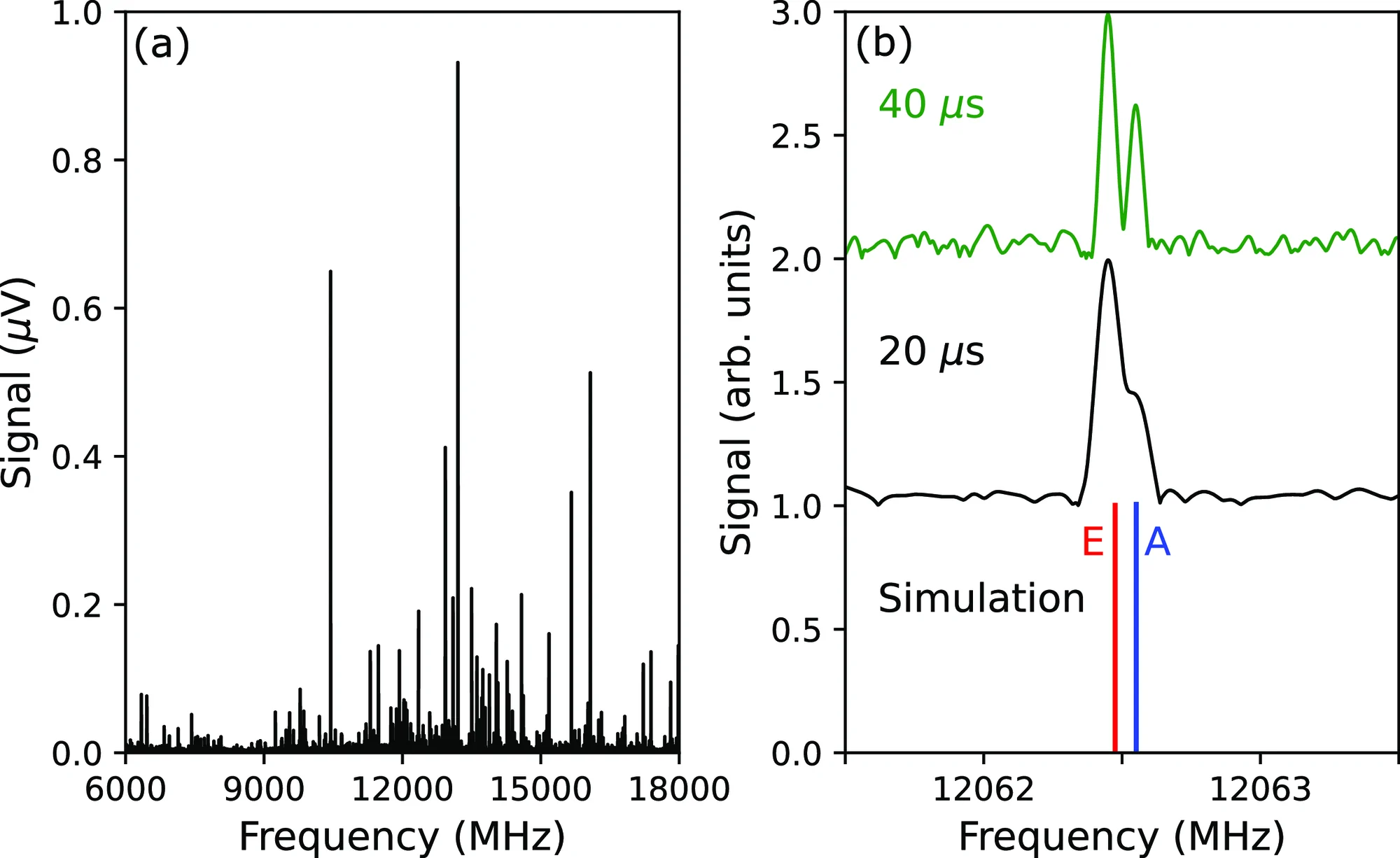
(a) 6–18 GHz spectrum of 2-MST obtained with 4 million averages in the Union spectrometer. The noise floor of approximately 0.004 μV results in a signal-to-noise ratio (SNR) of up to 230 for the strongest line. (b) Simulated spectrum (bottom) of the A (blue) and E (red) symmetry components of the 9_3,7_–9_2,7_ transition of 2-MST compared with experimental spectra obtained with a 20 μs (black, middle) or 40 μs (green, top) FID observation window.

A set of 26 *a*-type *R*-branch, 33 *b*-type *Q*-branch, and 53 *b*-type *R*-branch transitions were assigned to a single conformer of 2-MOT and results of the fit are shown in Table 1. Due to the lower signal-to-noise ratio of our spectra in the 75–100 GHz region, only the most intense *b*-type *R*-branch transitions were assigned in that spectral region. By comparison with rotational constants calculated at the M06–2X/def2-QZVP level of theory, this species is assigned to the *syn* conformation, in which the methoxy group is pointed *anti* to the sulfur heteroatom and *syn* to the C=C double bond. The coplanar orientation of the C–O and C=C bonds is further supported by calculation of the inertial defect (−2 × the planar moment of inertia along the *c*-axis). The value of – 3.35 amu·Å^2^ is consistent with a typical value of – 3.28 amu·Å^2^ for molecules with only two H atoms of a CH_2_ or CH_3_ group out-of-plane.^40^ Due to the larger data set for 2-MOT, this was the only species in which a full set of quartic centrifugal distortion constants could be obtained. Curiously, this is also the only molecule for which a significant discrepancy exists between the theoretical and experimental centrifugal distortion constants. At this level of theory, δ_*K*_ is predicted to be 1 order of magnitude smaller and Δ_*K*_ has the opposite sign. We also investigated this molecule at the ωB97X-D3/6–31+G­(d) level of theory,^41–43^ a combination suggested by the authors of ref 36. as a highly affordable strategy to obtain statistically robust rotational constant predictions. As shown in Table S4, the equilibrium rotational constants calculated at this level of theory are comparable to the vibrationally corrected rotational constants from the M06–2X/def2-QZVP calculations. Interestingly, VPT2 calculations at the ωB97X-D3/6–31+G­(d) level of theory produced centrifugal distortion constants, particularly Δ_*K*_ and δ_*K*_, in better agreement with the experimental determinations. A 1 order of magnitude difference in Δ_*K*_ between these theoretical methods was also found for 2-MST. Aside from these few examples, no significant difference was found for any other centrifugal distortion constants calculated by the two methods for these molecules.

**Table 1:** Experimental and Theoretical [M06-2X/def2-QZVP] Molecular Constants for 2-MOT, in MHz^b^

*A*, *I* ^*r*^	Experiment	Theory
*A*	4828.69047(33)	4844.30661
*B*	1809.42658(14)	1806.83433
*C*	1327.80019(17)	1330.76781
Δ_*J*_ × 10^3^	0.09321(99)	0.09037
Δ_*JK*_ × 10^3^	0.2927(48)	0.2008
Δ_*K*_ × 10^3^	0.6312(42)	–0.09368
δ_*J*_ × 10^3^	0.02491(62)	0.02113
δ_*K*_ × 10^3^	0.312(13)	0.04658
Range of *J* values	2–17	–
*N* ^a^	112/100	–
rms	0.006	–

a Number of lines in fit/Number of unique frequencies.

b Numbers in parentheses are 1σ uncertainties in units of the last reported digit.

One conformation each of 3-MOT and 3-MST were observed and both assigned to the *syn* conformation, in which the methoxy/methylthio group is again oriented *syn* to the double bond (Tables 2 and 3). The calculated higher energy conformations of both *anti*-3-MOT and *skew*-3-MST are substantially closer to the prolate limit (κ = −0.90 and −0.96, respectively) than the calculated lower energy *syn* conformations or the experimental results (κ = −0.85 and −0.88, respectively). Inertial defects of −3.31 amu·Å^2^ and −3.48 amu·Å^2^ for 3-MOT and 3-MST, respectively, confirm the presence of just two out-of-plane H atoms for both species. Additionally, the *skew* conformation of 3-MST would be expected to display a predominantly *c*-type spectrum, but only *a*-type and *b*-type transitions could be assigned.

**Table 2:** Experimental and Theoretical [M06-2X/def2-QZVP] Molecular Constants for 3-MOT, in MHz^c^

*A*, *I* ^*r*^	Experiment	Theory
*A*	5658.43678(67)	5696.30979
*B*	1571.41607(33)	1570.00023
*C*	1239.86635(32)	1241.73018
Δ_*J*_ × 10^3^	0.0822(36)	0.07372
Δ_*JK*_ × 10^3^	[0.04876]^a^	0.04876
Δ_*K*_ × 10^3^	[0.7530]	0.7530
δ_*J*_ × 10^3^	0.02230(59)	0.01510
δ_*K*_ × 10^3^	[0.1145]	0.1145
Range of *J* values	1–14	–
*N* ^b^	70/69	–
rms	0.010	–

a Values in square brackets were fixed to the theoretical values.

b Number of lines in fit/Number of unique frequencies.

c Numbers in parentheses are 1σ uncertainties in units of the last reported digit.

**Table 3:** Experimental and Theoretical [M06-2X/def2-QZVP] Molecular Constants for 3-MST, in MHz^c^

*A*, *I* ^*r*^	Experiment	Theory
*A*	4659.00835(67)	4663.23345
*B*	1151.92474(38)	1155.09087
*C*	929.48468(37)	932.04711
Δ_*J*_ × 10^3^	0.0567(28)	0.04653
Δ_*JK*_ × 10^3^	[0.08104]^a^	0.08104
Δ_*K*_ × 10^3^	[0.2498]	0.2498
δ_*J*_ × 10^3^	0.01296(73)	0.007103
δ_*K*_ × 10^3^	[0.04881]	0.04881
Range of *J* values	2–13	–
*N* ^b^	82/82	–
rms	0.013	–

a Values in square brackets were fixed to the theoretical values.

b Number of lines in fit/Number of unique frequencies.

c Numbers in parentheses are 1σ uncertainties in units of the last reported digit.

The spectrum of 2-MST exhibited a number of line splittings due to internal rotation of the methyl group. In order to more completely characterize this internal motion, additional data for a small number of transitions were collected with a 40 μs FID observation window, which reduced the fwhm line width to approximately 60 kHz at 10 GHz as shown in Figure 4b. The resolution of these experiments was sufficient to extract line centers for 31 A and 37 E symmetry individual lines, and 70 blended transitions. A global fit of the A and E components was performed using the XIAM code, which excluded blended transitions, and the results are presented in Table 4.^44^ XIAM implements the “combined axis method”,^45^ in which the internal rotation operator is set up in a coordinate system parallel to the symmetry axis of the methyl rotor, diagonalized, and then transformed back into the principal axis system. This approach results in fit parameters that are directly comparable to those from rigid rotor fits in the principal axis system and to internal rotation barriers from quantum chemistry calculations. Only *V*_3_ was fit as an internal rotation parameter, with the internal rotor rotational constant *F*_0_, and the angles between the rotor axis and the *a* and *b* inertial axes fixed to calculated values. The obtained rotational constants are in good agreement with those of the *skew* conformation, and a large inertial defect (−66 amu·Å^2^) confirms that the C–S bond of the methylthio group does not lie coplanar with the ring. The experimental *V*_3_ barrier of 6.98 kJ/mol is consistent with the theoretical value of 6.69 kJ/mol.

**Table 4:** Experimental and Theoretical [M06-2X/def2-QZVP] Molecular Constants for 2-MST, in MHz^c^

*A*, *I* ^*r*^	Experiment	Theory
*A*	3937.77840(85)	3956.0613
*B*	1282.96891(67)	1283.7113
*C*	1107.47250(45)	1108.3327
Δ_*J*_ × 10^3^	0.1334(44)	0.1204
Δ_*JK*_ × 10^3^	1.6904(98)	2.111
Δ_*K*_ × 10^3^	[−1.012]^a^	–1.012
δ_*J*_ × 10^3^	[0.01364]	0.01364
δ_*K*_ × 10^3^	–4.27(11)	–3.209
*F*_0_ (GHz)	[158.838]	158.838
*V*_3_ (kJ/mol)	6.9788(33)	6.693
∠(*i*,*a*) (°)	[66.2605]	66.2605
∠(*i*,*b*) (°)	[101.5575]	101.5575
Range of *J* values	2–14	–
*N*_*A*_/*N*_*E*_ ^b^	31/37	–
rms	0.012	–

a Values in square brackets were fixed to the theoretical values.

b Number of A and E component lines included in fit.

c Numbers in parentheses are 1σ uncertainties in units of the last reported digit.

### Conformational Preferences

For all four species studied here, only the lowest energy conformation was observed in our experiments. This is striking given the ZPE corrected energy differences of less than 4 kJ/mol between conformers of 2-MOT, 2-MST, and 3-MST. A second experiment in which the carrier gas was changed from argon to neon was performed on 3-MST. Due to its lower mass, neon was anticipated to be less efficient at cooling the conformational degrees of freedom. However, we again found no evidence of the higher energy *skew* conformation. Our nonobservations support a mechanism of efficient conformational cooling over low-energy barriers as observed in rare gas jets for other molecules.^46–48^ Indeed, the barriers for interconversion from the higher to lower energy conformations range from just 1.8 kJ/mol for 2-MST up to 8.5 kJ/mol for 3-MOT.

The experimentally observed conformations of 2- and 3-MOT are consistent with the geometries obtained at the M06–2X/def2-QZVP level of theory shown in Figure 5. In both molecules, the orientation of the methoxy substituent *syn* to the double bond is the preferred conformation. A second, higher-energy conformation of each molecule was identified with the methoxy substituent oriented *anti* to the double bond. The stronger conformational preference in 3-MOT for the *syn* orientation is notable since the substituent group is *meta* to the heteroatom and suggests that the sulfur atom itself does not play a “directing” role. The related species methyl vinyl ether also adopts a lowest energy conformation in which the C–O bond is coplanar and *syn* to a C=C bond.^49^ Similarly, anisole (methoxybenzene) exhibits bond length alternation in the benzene ring and the methoxy group lies *syn* to a shorter carbon–carbon bond.^50^

**Figure 5:**
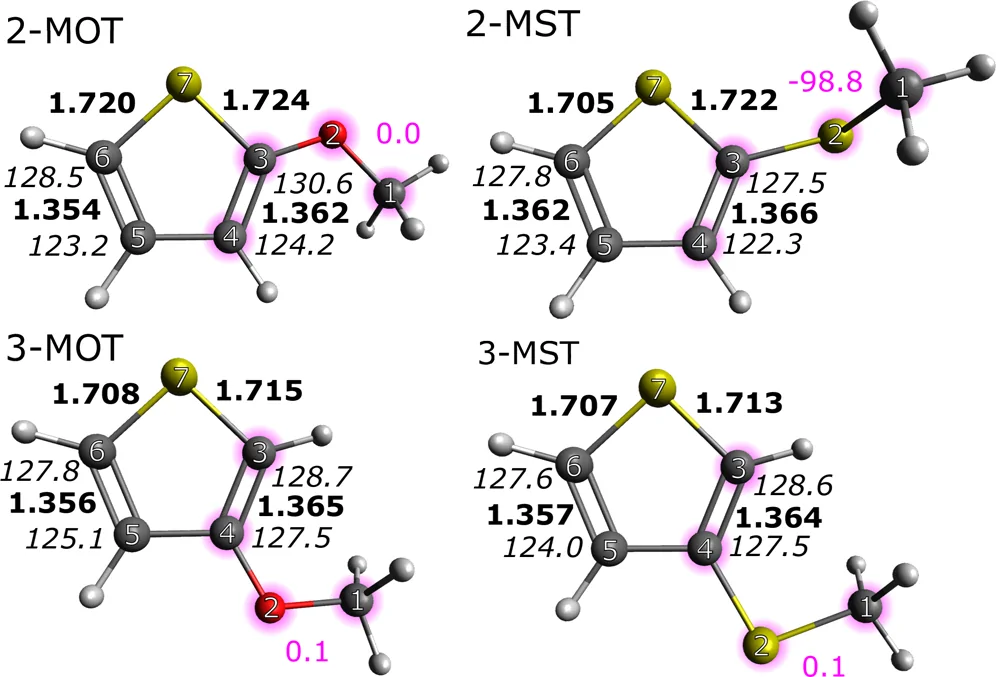
Geometries, atom numbering, and selected structural parameters calculated at the M06–2X/def2-QZVP level of theory. Bond lengths appear in bold text in units of Å. Bond angles appear in italic text in units of degrees. One dihedral angle appears in plain, magenta text for the four highlighted atoms.

In order to gain some insight into the origins of this conformational preference, we performed a natural bond orbital (NBO) analysis using NBO 7.0.^51^ Table 5 presents a summary of these results. The difference in energy due to resonance stabilization was estimated as the sum of all second order perturbation theory energy corrections between the OCH_3_ group and the thiophene ring. We further divided this stabilization into conjugative contributions, specifically the O­(2p) → C = C­(π*) donation, and hyperconjugative contributions involving donation into σ* orbitals. In both 2-MOT and 3-MOT, the conjugation strongly favors the preferred *syn* conformation by 14.8 and 15.0 kJ/mol, respectively. The difference in energy due to O­(2p) → C=C­(π*) conjugation correlates with a geometric difference between the two conformers. As shown in Figure 6a, both preferred conformations adopt a larger C=C–O angle than the higher energy conformations. To further examine this effect, we performed NBO analysis while scanning the C4=C3–O2 angle of *syn*-2-MOT with all other geometric parameters fixed to the previously optimized geometry. In Figure 6b, the second order energy correction due to O2­(2p) → C3=C4­(π*) conjugation is plotted on the left-hand axis showing a general increase as the C4=C3–O2 angle increases up to 155°. Simultaneously, the C3=C4 π bond occupancy is plotted on the right-hand axis in Figure 6b and reveals a similar increase. As the C4=C3–O2 angle increases, the electrostatic repulsion between the O­(2p) lone pair and the C3=C4 π bond electron density is expected to decrease. This then allows for increased electron density to be accommodated at the double bond as shown by both the larger energy correction due to conjugation and the larger C=C π bond occupancy. Increased conjugation appears to be the dominant factor favoring the *syn* conformations in 2-MOT and 3-MOT. A similar effect is noted by Bond and Schleyer in their analysis of acyclic unsaturated ethers.^52^ They similarly conclude that the wider angle reduces electronic repulsion and allows for greater occupancy of the delocalized molecular orbital centered around the O–C=X moiety.

**Figure 6:**
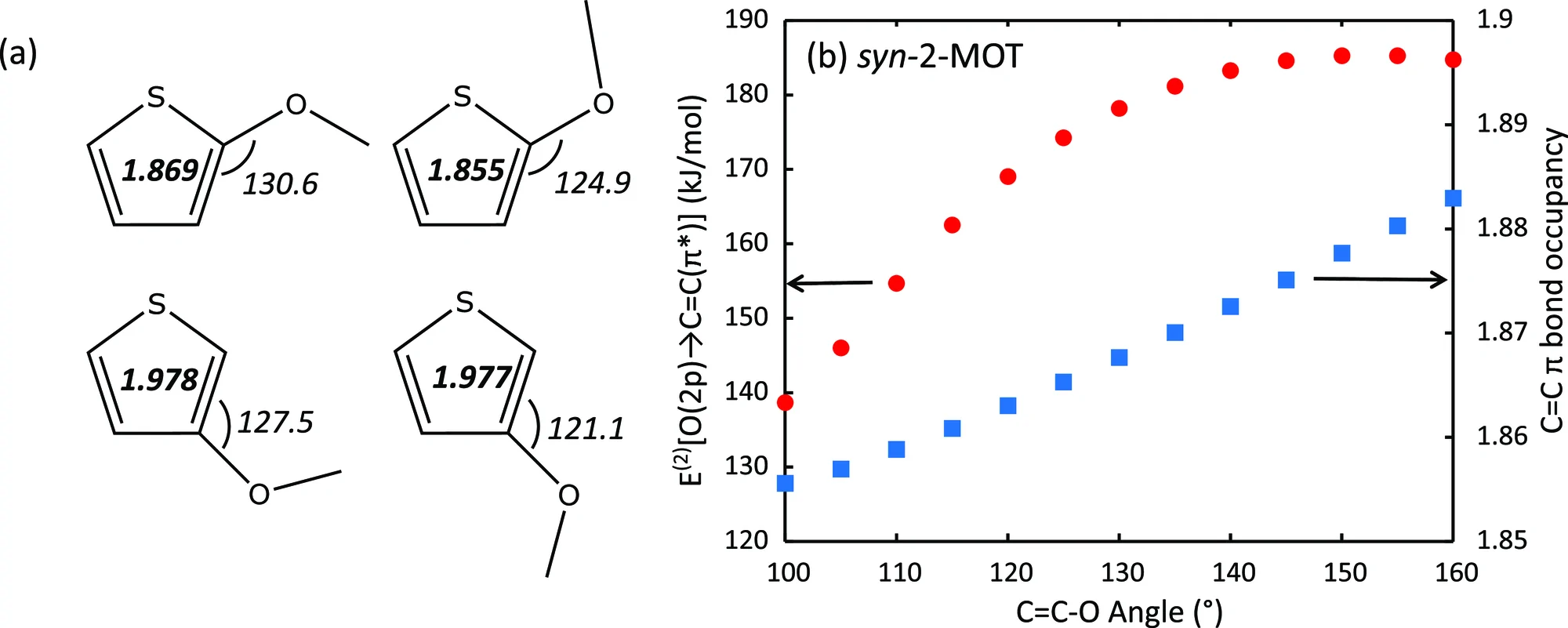
(a) Two conformations of 2-MOT and 3-MOT with the C=C–O angle (italics) and C=C π bond occupancy (bold italics) indicated. Structures were optimized at the M06–2X/def2-QZVP level of theory. (b) Left axis: Magnitude of the second order energy correction (red circles) due to O­(2p) → C =C­(π*) conjugation as a function of C=C–O angle. Right axis: The C=C π bond occupancy (blue squares) as a function of C=C–O angle. All other geometric parameters are fixed to the values from the optimized geometry of *syn*-2-MOT.

**Table 5:** Energy Differences between the Two Conformers of 2-MOT, 3-MOT, 2-MST, and 3-MST in kJ/mol[Table-fn t5fn1]

	2-MOT	3-MOT	2-MST	3-MST
Δ*E*_conj_	–14.8	–15.0	–112.1	–113.0
Δ*E*_hyper_	–7.5	–2.1	82.0	64.7
Δ*E*_steric_	8.3	8.5	4.1	22.0
Δ*E*_sum_	–13.9	–8.6	–26.0	–26.3
Δ*E*	–3.5	–7.9	2.9	–4.1

1 In all cases, a negative energy indicates a preference for the *syn* conformer and a positive energy indicates a preference for the second (*anti* or *skew*) conformer. First and second row: Differences between the second order perturbation theory energy corrections for conjugative and hyperconjugative interactions. Third row: Differences in pairwise steric exchange energies. Fourth row: Sum of the energy differences due to conjugation, hyperconjugation, and sterics; Δ*E*_sum_ = Δ*E*_conj_ + Δ*E*_hyper_ + Δ*E*_steric_. Fifth row: The electronic energy difference between conformers calculated at the M06-2X/def2-QZVP level of theory.

The energy difference due to hyperconjugation also favors the preferred *syn* conformations of both species and involves four contributions. The largest hyperconjugative interaction is the O­(*n*) → C–X­(σ*) interaction between the lone pair in a hybrid orbital on oxygen and the antibonding orbital of the bond lying antiperiplanar. This hyperconjugation can be described as a type of anomeric effect at *sp* ^2^ centers and is typically invoked to explain the well-known *syn* preference of esters and carboxylic acids.^53^ Bond and Schleyer discuss similar stereoelectronic effects in their analysis of methyl formate, vinyl alcohol and methyl vinyl ether, which all similarly prefer a *syn* conformation.^52^ These authors point out that donation from the “back” lobe of the hybrid orbital on O can also contribute to the hyperconjugation, and, indeed we find that the sum of the O­(*n*) → C–X­(σ*) interactions with both neighboring bonds is essentially unchanged between the *anti* and *syn* conformations reflecting donation from both the “front” and “back” lobes of the lone pair hybrid orbital. The second set of hyperconjugative contributions in *syn*-2-MOT are the C1–O2­(σ) → C3– S7­(σ*) and C3–S7­(σ) → C1–O2­(σ*) interactions. In *anti*-2-MOT the equivalent interactions occur between the C1–O2 and C3–C4 bonds. Due to the higher electronegativity of sulfur relative to carbon, the orbital energy difference for the *syn*-2-MOT interactions is smaller resulting in larger energy corrections and thus the energetic preference for *syn* over *anti*. In contrast, the equivalent σ → σ* hyperconjugative interactions for *syn*- and *anti*-3-MOT involve the C3–C4 and C4–C5 bonds, respectively. Thus, there is a smaller difference in the energy corrections due to hyperconjugation between the two conformers of 3-MOT.

The pairwise steric exchange energy was also calculated with NBO 7.0 for the two conformers of 2-MOT and 3-MOT. The difference in energy due to steric repulsion was estimated as the sum of all pairwise steric exchange energies between the OCH_3_ group and the thiophene ring. In both molecules, the *anti* conformer is calculated to have less steric repulsion between these two moieties than the *syn* conformer. In 2-MOT, this is predominantly due to the greater steric exchange energy between the methyl group and the C4–H bond in the *syn* conformation. In 3-MOT, the repulsion between the O­(2p) lone pair and C3=C4­(π) bond is larger in the *syn* conformation, presumably because of the greater electron density at C3=C4 as discussed above, and in spite of the wider C3=C4–O2 angle in this conformation.

The sum of these orbital and steric contributions correctly predicts the lower energy conformations of 2-MOT and 3-MOT, shown in Table 5. However, the stability of *syn*-2-MOT is overestimated from this simple energy decomposition analysis. Electrostatic contributions have not been explicitly considered here and may play an important role. In particular, in many cases, conformational preferences of molecules composed of two distinct moieties have been attributed to an electrostatic interaction of dipoles. When the dipoles of each moiety are oriented antiparallel, the molecular dipole is reduced and the total energy is lowered. This principle correctly predicts the lower energy conformer of methyl formate, methyl vinyl sulfide, vinyl alcohol, and other molecules.^10,52^ In *syn*-2-MOT, the dipoles of the thiophene ring and methoxy substituent are oriented nearly parallel and the calculated dipole moment is about twice that of *anti*-2-MOT. This dipole–dipole energy might be thus expected to decrease the energetic preference for *syn*-2-MOT, and explains the modest electronic energy difference between these two conformations. The situation is reversed for 3-MOT, where the preferred orientation of dipoles reinforces the energetic preference for the *syn* conformer. However, the stability of *syn*-3-MOT relative to *anti* is already slightly overestimated from steric and orbital considerations alone, highlighting the limitations of the physically insightful, but simple models considered here.

The conformational preferences of the 2- and 3-MST differ from those of 2- and 3-MOT primarily due to the weaker donation of the methylthio S2­(3*p*) lone pair electrons into the ring. The second order perturbation theory corrections for this interaction in *syn*-2-MST and *syn*-3-MST are 30–40% smaller relative to the methoxy-substituted analogues. In the *skew* conformations, conjugation is disallowed due to the orientation of orbitals, which results in a large conjugative stabilization of the *syn* conformations relative to *skew*. Moreover, the C4=C3–S2 angle in 2-MST is identical to the C5=C6–H angle (Figure 5); no distortion of the C4=C3–S2 angle is present for *skew*-2-MST due to the absence of both *p*–π repulsion and subsequent stabilizing *p* → π* donation. The same angle dependent resonance stabilization discussed for the MOT species contributes to the lower energy of the *syn* conformers of 2-MST and 3-MST relative to the *anti* geometries, which now appear as saddle points on the potential energy curves. This energy difference combined with the position of the well for the *skew* conformation geometrically closer to the *anti* than the *syn* structure eliminates the *anti* minimum from the potential energy curves of MST.

The second order energy corrections due to hyperconjugation favor the *skew* conformers due to effective donation of both the S2­(*n*) and S2­(3*p*) lone pairs into the neighboring C3–S7 and C3–C4 σ* orbitals for 2-MST or C3–C4 and C4–C5 σ* orbitals for 3-MST. As in the MOT species, the energy difference due to hyperconjugation is greater for 2-MST than 3-MST because of the smaller energy difference between the S2 lone pairs and the C3–S7 σ* orbital relative to a carbon–carbon σ* orbital. The sum of conjugative and hyperconjugative energies alone thus results in a stronger *syn* preference for the 3-MST over the 2-MST.

Chemical intuition suggests that the steric repulsion between the methylthio substituent and the thiophene ring would be substantially reduced in a *skew* conformation relative to one in which the C1–S2 bond lies coplanar with the thiophene ring. The sum of pairwise steric exchange energies calculated with NBO 7.0 leads to an energy difference of 22.0 kJ/mol for 3-MST, but just 4.1 kJ/mol for 2-MST. In the case of *skew*-2-MST, large steric exchange energies are calculated for the interaction of the S2­(3*p*) lone pair with the neighboring C3–S7 and C3–C4 σ bonds, as well as between the C1–S2 σ bond and the C3–S4 π bond, which offset the reduction in S2­(3*p*) −C3=C4­(π) repulsion. As a result, the sum of steric and orbital energy differences predicts a strong preference for the *syn* conformer for 2-MST. While the difference in steric exchange energies is larger for 3-MST relative to 2-MST, the energetic preference for *syn* is also overestimated for this molecule.

In addition to NBO analysis, we considered the intramolecular interaction between the methylthio group and thiophene ring using the noncovalent interaction (NCI) index analysis.^54^ NCI is a topological method that identifies noncovalent interactions as regions where the electron density gradient is sharply reduced. The strength of such interactions scales with the electron density and the sign of the second eigenvalue of the density gradient indicates whether the interaction is attractive or repulsive. The results of NCI analysis for the conformers of 2-MOT and 2-MST are shown in Figure 7. The small red surface at the center of the thiophene ring may be interpreted as ring strain, while the green surfaces indicate weak interactions with both attractive and repulsive contributions. The similar color and area of the NCI surfaces for *syn* and *anti*-2-MOT indicate similar noncovalent interactions between the methoxy subsituent and thiophene ring for both species. In contrast, the NCI analysis of 2-MST reveals similarly weak attractive and repulsive interactions in the *syn* conformer, but much weaker interactions in the *skew* conformer, not visible with the isosurface value set in Figure 7. Similar results were found for 3-MOT and 3-MST. These contrasting NBO and NCI results suggest that the difference in steric repulsion may be underestimated or the difference in resonance stabilization overestimated in the NBO analysis. Both possibilities have been raised in similar studies of challenging small molecule systems.^55–57^ In addition, the dipole–dipole interaction previously discussed would be expected to destabilize *syn*-2-MST relative to *skew* and stabilize *syn*-3-MST relative to *skew*, however, the energetic difference should be smaller than in the MOT conformers due to the out-of-plane orientation of the dipoles in the *skew* conformers. In the future, more sophisticated energy decomposition techniques^58–60^ may help to shed more light on the subtle energetic contributions responsible for the conformational preferences in these molecules.

**Figure 7:**
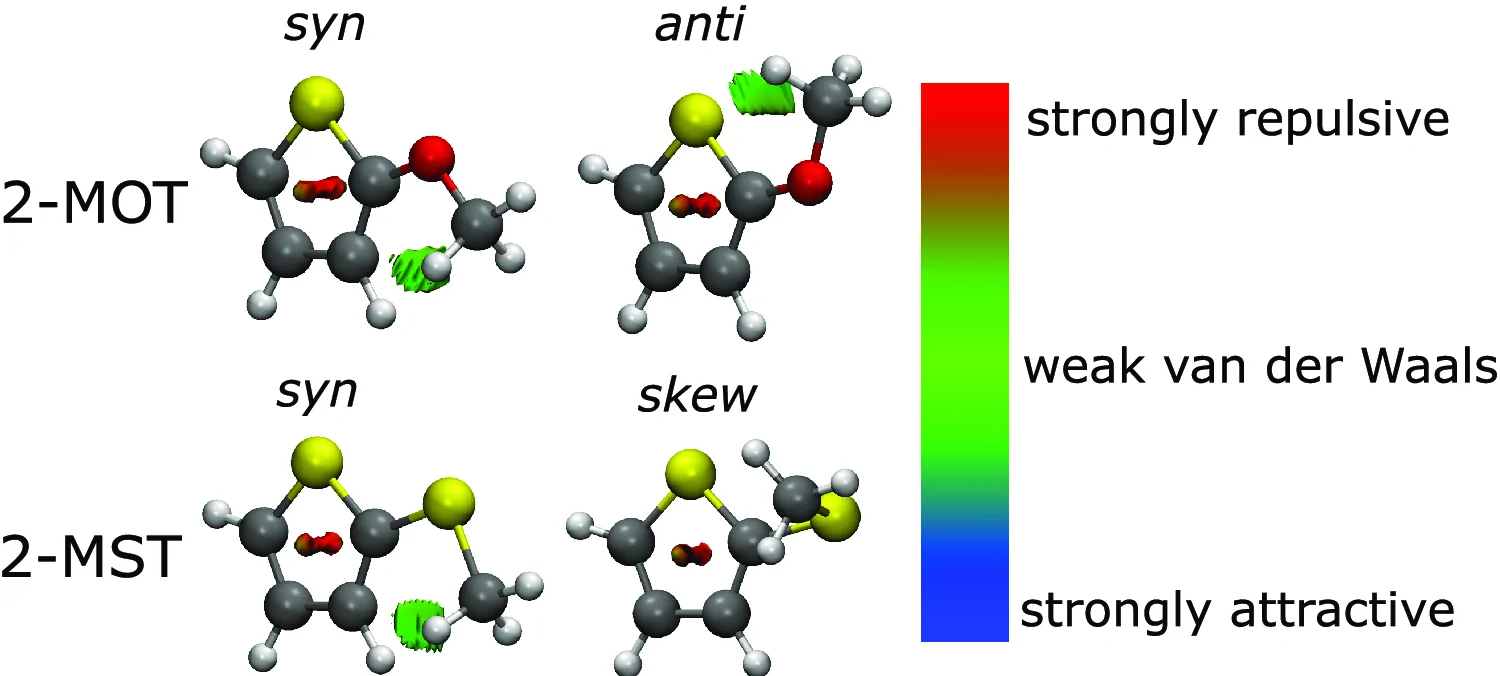
NCI plots for the two conformations of 2-MOT and 2-MST. Strong steric repulsions at the center of each thiophene ring are visualized in red. Weak interactions with repulsive and attractive contributions between the methyl group and the ring are shown as the small green surface for all species except *skew*-2-MST.

## Conclusion

Using a newly constructed 6–18 GHz segmented CP-FTMW spectrometer, we have characterized the rotational spectra of the four substituted thiophenes 2-MOT, 3-MOT, 2-MST, and 3-MST. Under the jet-cooled conditions of the experiment, efficient conformational cooling leads to the observation of only the lowest energy conformation of each species. In three of the four molecules, this conformation orients the methoxy or methylthio substituent *syn* to the double bond with the C–O/C–S bond of the substituent coplanar with the thiophene ring. NBO analysis reveals both *p*–π*conjugative and *n*–σ*hyperconjugative interactions between the ring and substituent group are responsible for the preferred planarity, while a wider C=C–X bond angle adopted in the *syn* conformations allows for greater donation to the π system and favors this orientation over the *anti*. The preferred 2-MST conformer has an out-of-plane *skew* orientation of the methylthio group, which reduces steric interactions. In this unique conformation, the barrier to methyl internal rotation is substantially lowered, resulting in experimentally resolvable splittings. Given the significance of subtle geometric changes in explaining the conformational preferences of these molecules, experimental structure determination is highly desirable. Future improvements to our newly built spectrometer are anticipated to make observation of rare isotopologues feasible, enabling such analysis. This study indicates that substitution positions *ortho* and more distant from the heteroatom can be leveraged for conformational directing in functionalized heterocycles and the rational design of three-dimensional molecular structure.

## Supplementary Material







## Notes

Optimized M06–2X/def2-QZVP geometries in xyz format (ZIP)
PGOPHER and XIAM input files for all fits described in the text (ZIP)
Complete component list for the CP-FTMW spectrometer; figures of methyl torsion potential energy curves; results for *S*-reduction fits; ωB97X-D3/6–31+G­(d) molecular constants; tables listing second order energy corrections and pairwise steric exchange energies from the NBO analysis; complete experimental line lists (PDF)

## †

Department of Physics, New York University, New York, New York 10003, United States
