# Supplementary material for: Conformational Analysis of Methoxy- and Methylthio-Substituted Thiophenes by Broadband Rotational Spectroscopy and Computational Chemistry
Source: J Phys Chem A. 2026 Oct 8;130(42):8268–78. doi: 10.1021/acs.jpca.6c04394 (PMC13700099; doi:10.1021/acs.jpca.6c04394)
Supplement: Supplementary file 1 [file jp6c04394_si_003.pdf]

Supporting Information: Conformational Analysis of Methoxy- and Methylthio-Substituted Thiophenes by Broadband Rotational Spectroscopy and Computational Chemistry

Timothy J. Barnum,*,† Nathan Riordon,† Lukas Baker,†,‡ and Nicholas J. Gestring†

†*Department of Chemistry, Union College, Schenectady, NY 12308, USA*

‡*Current address: Department of Physics, New York University, New York, NY 10003, USA*

E-mail: barnumt@union.edu

Component list for 6–18 GHz CP-FTMW spectrometer as labeled in Figure 1.

i. 10 MHz Rubidium frequency standard (Stanford Research Systems, FS725)

ii. PXIe Arbitrary waveform generator, 1 GS/s (Keysight, M3202A)

iii. Frequency synthesizer, 0.01–20 GHz (Valon, 5019)

iv. Power divider, -3 dB, 2–26.5 GHz (Mini-Circuits, ZC2PD-02263-S+)

v. Double-balanced mixer, 5–20 GHz (Narda Miteq, DM0520LW1)

vi. Power amplifier, 6–18 GHz, 40 W (RF-Lambda, RFLUPA0618GC)

vii. Fixed attenuator, -10 dB, 50 W (Mini-Circuits, BW-N10W50+)

viii. Triple-balanced mixer, 2–18 GHz, -10 dB (Macom, M93)

ix. SPST PIN Switch, 10 ns, 2–18 GHz, 3 W (Narda Miteq, S213D)

x. Quad ridged, dual polarization horn antenna, 4-40 GHz (RFSpin, QRH40)

xi. Diode limiter, 1–18 GHz (Narda Miteq, LIM 101)

xii. Low noise amplifier, +40 dB, 6–18 GHz (Narda Miteq, LNA-40-06001800-15-10P)

xiii. Low pass filter, DC–400 MHz (Mini-Circuits, SLP-450+)

xiv. DC Block, 0.001–8 GHz (Mini-Circuits, BLK-89-S+)

xv. PXIe Digitizer, 3.2 GS/s, 500 MHz (Keysight, M9203A)

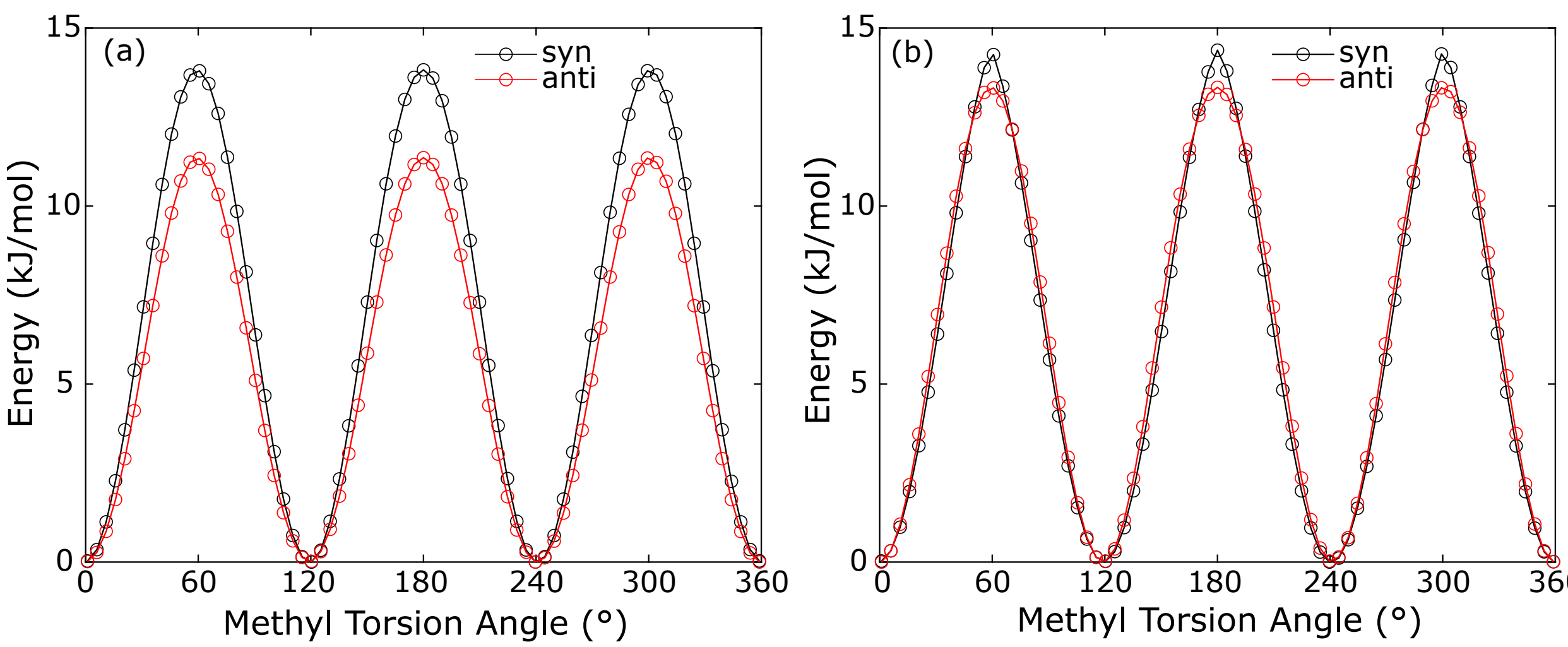

Figure S1: Potential energy surface of (a) 2-MOT and (b) 3-MOT calculated at the M06-2X/def2-QZVP level of theory along the methyl torsional coordinate. The *syn* conformations are shown in black and have slightly higher barriers to internal rotation relative to the *anti* conformations shown in red.

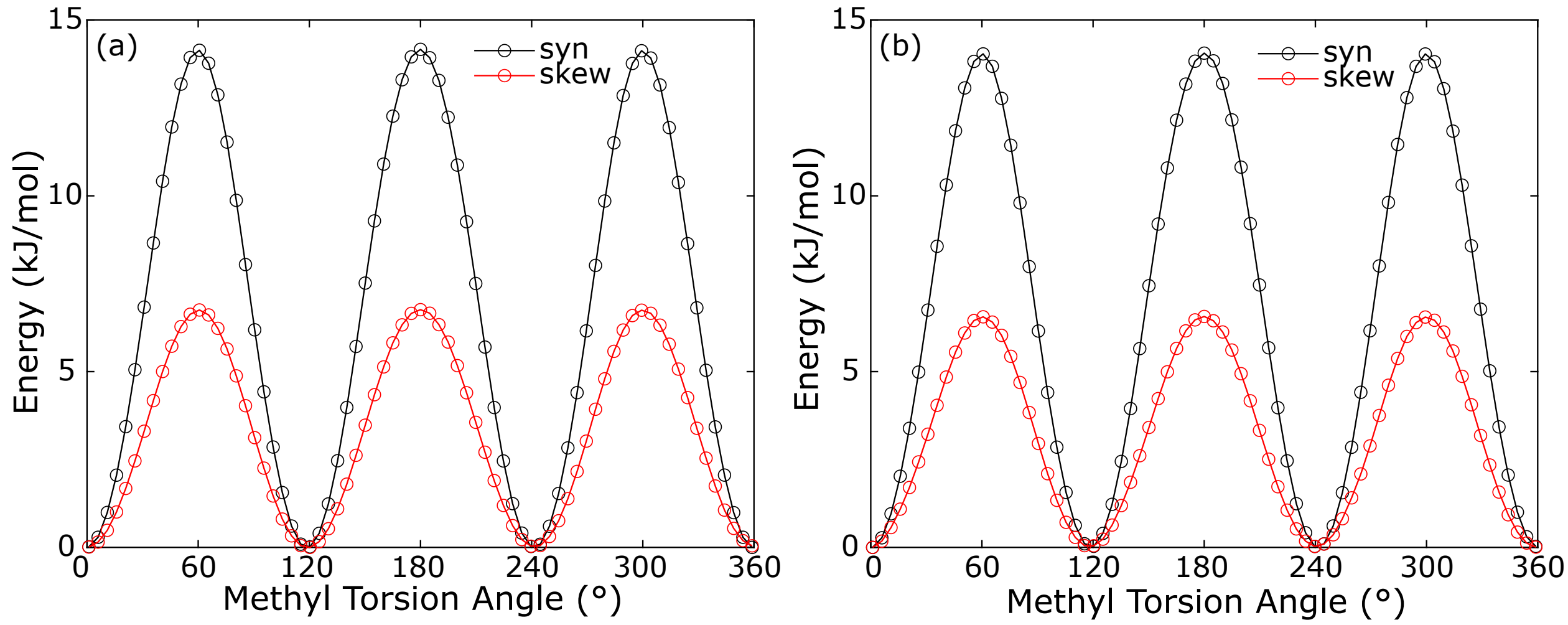

Figure S2: Potential energy surface of (a) 2-MST and (b) 3-MST calculated at the M06-2X/def2-QZVP level of theory along the methyl torsional coordinate. The *skew* conformations, shown in red, exhibit significantly lower barriers to internal rotation relative to the *syn* conformations shown in black.

Table S1: Experimental and theoretical [M06-2X/def2-QZVP] molecular constants for 2-MOT, in MHz, for an S reduction fit. Numbers in parentheses are 1σ uncertainties in units of the last reported digit.

S, I^r	This work	Theory
A	4828.69053(33)	4844.30661
B	1809.42593(14)	1806.83433
C	1327.80079(18)	1330.76781
$D_J \times 10^3$	0.08169(76)	0.08846
$D_{JK} \times 10^3$	0.3618(33)	0.2122
$D_K \times 10^3$	0.5735(29)	-0.1032
$d_1 \times 10^3$	-0.02491(62)	-0.02113
$d_2 \times 10^3$	-0.00576(23)	-0.0009549
Range of J values	2–17	–
N[a]	112/100	–
rms	0.006	–

[a] Number of lines in fit/Number of unique frequencies.

Table S2: Experimental and theoretical [M06-2X/def2-QZVP] molecular constants for 3-MOT, in MHz, for an S reduction fit. Numbers in parentheses are 1σ uncertainties in units of the last reported digit.

S, I^r	This work	Theory
A	5658.43636(65)	5696.30979
B	1571.41596(33)	1570.00023
C	1239.86649(31)	1241.73018
$D_J \times 10^3$	0.0806(35)	0.07190
$D_{JK} \times 10^3$	[0.05967]^a	0.05967
$D_K \times 10^3$	[0.7439]	0.7439
$d_1 \times 10^3$	-0.02293(58)	-0.01510
$d_2 \times 10^3$	[-0.0008917]	-0.0008917
Range of J values	1–14	–
N^b	70/69	–
rms	0.010	–

^{a} Values in square brackets were fixed to the ab initio values. ^{b} Number of lines in fit/Number of unique frequencies.

Table S3: Experimental and theoretical [M06-2X/def2-QZVP] molecular constants for 3-MST, in MHz, for an S reduction fit. Numbers in parentheses are 1σ uncertainties in units of the last reported digit.

S, I^r	This work	Theory
A	4659.00833(67)	4663.23345
B	1151.92468(38)	1155.09087
C	929.48473(37)	932.04711
$D_J \times 10^3$	0.0559(28)	0.04600
$D_{JK} \times 10^3$	[0.07180]^a	0.08423
$D_K \times 10^3$	[0.2520]	0.2472
$d_1 \times 10^3$	-0.01318(73)	-0.007103
$d_2 \times 10^3$	[-0.0002085]	-0.0002652
Range of J values	2–13	–
N^b	82/82	–
rms	0.013	–

^{a} Values in square brackets were fixed to the ab initio values. ^{b} Number of lines in fit/Number of unique frequencies.

Table S4: Theoretical [ωB97X-D/6-31+G(d)] molecular constants for the observed conformations of 2-MOT, 3-MOT, 2-MST and 3-MST, in MHz, for an A reduction.

A, I^r	2-MOT	3-MOT	2-MST	3-MST
A_e	4836.55172	5684.06500	3930.27912	4653.67833
A_0	4814.66688	5655.28493	3912.29158	4630.59431
B_e	1808.48107	1565.81601	1269.92085	1145.80677
B_0	1792.75890	1551.42597	1263.02563	1135.31404
C_e	1327.48100	1237.54327	1100.5381	924.85973
C_0	1317.88765	1228.24970	1094.24247	918.26430
$\Delta_J \times 10^3$	0.0865	0.07451	0.09802	0.04550
$\Delta_{JK} \times 10^3$	0.2797	0.03128	1.185	0.06930
$\Delta_K \times 10^3$	0.5852	0.7720	-0.1465	0.2541
$\delta_J \times 10^3$	0.02445	0.01535	0.008086	0.006955
$\delta_K \times 10^3$	0.302	0.1138	-3.494	0.03924

Table S5: Second order perturbation theory energy corrections to the NBO energy, in units of kJ/mol, attributed to conjugation between the methoxy/methylthio substituent and the thiophene ring for both conformers of 2-MOT, 3-MOT, 2-MST, and 3-MST.

2-MOT					
syn			*anti*		
LP (2) O 2	BD*(2) C 3- C 4	180.7	LP (2) O 2	BD*(2) C 3- C 4	165.9
3-MOT					
syn			*anti*		
LP (2) O 2	BD*(2) C 3- C 4	196.3	LP (2) O 2	BD*(2) C 3- C 4	181.3
2-MST					
syn			*skew*		
LP (2) S 2	BD*(2) C 3- C 4	121.4	LP (1) S 2	BD*(2) C 3- C 4	9.3
3-MST					
syn			*skew*		
LP (2) S 2	BD*(2) C 3- C 4	135.7	LP (2) S 2	BD*(2) C 3- C 4	5.4

Table S6: Second order perturbation theory energy corrections to the NBO energy, in units of kJ/mol, attributed to hyperconjugation between the methoxy substituent and the thiophene ring for both conformers of 2-MOT and 3-MOT.

2-MOT					
syn			*anti*		
LP (1) O 2	BD*(1) C 3- C 4	40.2	LP (1) O 2	BD*(1) C 3- C 4	3.8
LP (1) O 2	BD*(1) C 3- S 7	9.2	LP (1) O 2	BD*(1) C 3- S 7	44.3
BD (1) C 1- O 2	BD*(1) C 3- S 7	17.9	BD (1) C 1- O 2	BD*(1) C 3- C 4	14.6
BD (1) C 3- S 7	BD*(1) C 1- O 2	14.5	BD (1) C 3- C 4	BD*(1) C 1- O 2	11.5
3-MOT					
syn			*anti*		
LP (1) O 2	BD*(1) C 4- C 5	5.1	LP (1) O 2	BD*(1) C 4- C 5	39.2
LP (1) O 2	BD*(1) C 3- C 4	38.2	LP (1) O 2	BD*(1) C 3- C 4	4.2
BD (1) C 4- C 5	BD*(1) C 1- O 2	12.2	BD (1) C 3- C 4	BD*(1) C 1- O 2	10.8
BD (1) C 1- O 2	BD*(1) C 4- C 5	15.4	BD (1) C 1- O 2	BD*(1) C 3- C 4	14.6

Table S7: Second order perturbation theory energy corrections to the NBO energy, in units of kJ/mol, attributed to hyperconjugation between the methylthio substituent and the thiophene ring for both conformers of 2-MST and 3-MST.

2-MST					
syn			*skew*		
LP (1) S 2	BD*(1) C 3- C 4	20.8	LP (1) S 2	BD*(1) C 1- H 11	3.4
BD (1) C 1- S 2	BD*(1) C 3- S 7	15.6	LP (1) S 2	BD*(1) C 3- C 4	5.5
			LP (1) S 2	BD*(1) C 3- S 7	6.1
			LP (2) S 2	BD*(1) C 3- C 4	28.0
			LP (2) S 2	BD*(1) C 3- S 7	39.0
			LP (2) S 2	BD*(1) C 6- S 7	3.4
			BD (1) C 1- S 2	BD*(2) C 3- C 4	17.2
			BD (2) C 3- C 4	BD*(1) C 1- S 2	15.9
3-MST					
syn			*skew*		
LP (1) S 2	BD*(1) C 3- C 4	19.7	LP (1) S 2	BD*(1) C 4- C 5	12.9
BD (1) C 4- C 5	BD*(1) C 1- S 2	2.6	LP (1) S 2	BD*(2) C 3- C 4	6.7
BD (1) C 1- S 2	BD*(1) C 4- C 5	11.6	LP (2) S 2	BD*(1) C 3- S 7	3.2
			LP (2) S 2	BD*(1) C 4- C 5	19.5
			LP (2) S 2	BD*(1) C 3- C 4	23.2
			BD (2) C 3- C 4	BD*(1) C 1- S 2	12.8
			BD (1) C 1- S 2	BD*(1) C 3- C 4	4.0
			BD (1) C 1- S 2	BD*(2) C 3- C 4	16.4

Table S8: Pairwise steric exchange energies, in units of kJ/mol, for interactions between the methoxy substituent and the thiophene ring in both conformers of 2-MOT and 3-MOT.

2-MOT

syn			*anti*		
LP (1) O 2	BD (1) C 3- C 4	26.0	LP (1) O 2	BD (1) C 3- C 4	15.0
LP (1) O 2	BD (1) C 3- S 7	21.7	LP (1) O 2	BD (1) C 3- S 7	29.6
LP (2) O 2	LP (2) S 7	8.8	LP (2) O 2	LP (2) S 7	6.9
LP (2) O 2	BD (2) C 3- C 4	68.7	LP (2) O 2	BD (2) C 3- C 4	69.2
LP (2) O 2	BD (2) C 5- C 6	6.1	LP (2) O 2	BD (2) C 5- C 6	6.2
BD (1) C 1- O 2	BD (1) C 3- C 4	14.6	LP (1) S 7	BD (1) C 1- H 12	4.2
BD (1) C 1- O 2	BD (1) C 3- S 7	23.4	LP (1) S 7	BD (1) C 1- H 13	4.2
BD (1) C 1- H 12	BD (1) C 3- C 4	2.5	BD (1) C 1- O 2	BD (1) C 3- C 4	16.0
BD (1) C 1- H 12	BD (1) C 4- H 10	5.0	BD (1) C 1- O 2	BD (1) C 3- S 7	16.6
BD (1) C 1- H 13	BD (1) C 3- C 4	2.6	BD (1) C 1- H 11	BD (1) C 3- S 7	2.1
BD (1) C 1- H 13	BD (1) C 4- H 10	5.1	BD (1) C 1- H 12	BD (1) C 3- S 7	3.1
			BD (1) C 1- H 12	BD (1) C 3- S 7	3.1

3-MOT

syn			*anti*		
LP (2) S 7	LP (2) O 2	10.3	LP (2) S 7	LP (2) O 2	12.6
LP (1) O 2	BD (1) C 4- C 5	22.5	LP (1) O 2	BD (1) C 4- C 5	28.2
LP (1) O 2	BD (1) C 3- C 4	25.6	LP (1) O 2	BD (1) C 3- C 4	18.5
LP (2) O 2	BD (2) C 5- C 6	5.8	LP (2) O 2	BD (2) C 5- C 6	4.8
LP (2) O 2	BD (2) C 3- C 4	87.4	LP (2) O 2	BD (2) C 3- C 4	77.8
BD (1) C 4- C 5	BD (1) C 1- O 2	19.0	BD (1) C 4- C 5	BD (1) C 1- O 2	18.7
BD (1) C 3- C 4	BD (1) C 1- O 2	17.1	BD (1) C 4- C 5	BD (1) C 1- H 11	2.8
BD (1) C 3- C 4	BD (1) C 1- H 11	2.8	BD (1) C 4- C 5	BD (1) C 1- H 13	2.8
BD (1) C 3- C 4	BD (1) C 1- H 13	2.8	BD (1) C 5- H 9	BD (1) C 1- H 11	5.4
BD (1) C 3- H 8	BD (1) C 1- H 11	4.9	BD (1) C 5- H 9	BD (1) C 1- H 13	5.5
BD (1) C 3- H 8	BD (1) C 1- H 13	4.8	BD (1) C 3- C 4	BD (1) C 1- O 2	17.5

Table S9: Pairwise steric exchange energies, in units of kJ/mol, for interactions between the methylthio substituent and the thiophene ring in both conformers of 2-MST and 3-MST.

2-MST					
syn			*skew*		
LP (1) S 2	BD (1) C 3- C 4	5.5	LP (1) S 2	BD (2) C 3- C 4	10.9
LP (1) S 2	BD (1) C 3- S 7	4.8	LP (2) S 2	BD (1) C 3- C 4	21.3
LP (2) S 2	LP (2) S 7	5.4	LP (2) S 2	BD (1) C 3- S 7	24.8
LP (2) S 2	BD (2) C 3- C 4	44.4	LP (2) S 2	BD (1) C 4- H 10	4.3
LP (2) S 2	BD (2) C 5- C 6	6.5	LP (2) S 7	BD (1) C 1- S 2	2.7
BD (1) C 1- S 2	BD (1) C 3- C 4	9.2	BD (1) C 1- S 2	BD (2) C 3- C 4	25.8
BD (1) C 1- S 2	BD (1) C 3- S 7	14.1	BD (1) C 1- S 2	BD (2) C 5- C 6	2.1
BD (1) C 1- H 12	BD (1) C 4- H 10	4.2	BD (1) C 1- H 12	BD (2) C 3- C 4	2.8
BD (1) C 1- H 13	BD (1) C 4- H 10	4.7			
3-MST					
syn			*skew*		
LP (2) S 7	LP (2) S 2	9.4	LP (1) S 2	BD (1) C 4- C 5	2.1
LP (1) S 2	BD (1) C 4- C 5	7.3	LP (1) S 2	BD (2) C 3- C 4	6.8
LP (1) S 2	BD (1) C 3- C 4	5.2	LP (2) S 2	BD (1) C 4- C 5	19.3
LP (2) S 2	BD (2) C 5- C 6	2.8	LP (2) S 2	BD (1) C 5- H 9	2.8
LP (2) S 2	BD (2) C 3- C 4	47.8	LP (2) S 2	BD (1) C 3- C 4	16.3
BD (1) C 4- C 5	BD (1) C 1-S 2	11.5	LP (2) S 2	BD (2) C 3- C 4	9.1
BD (1) C 3- C 4	BD (1) C 1-S 2	11.0	LP (2) S 2	BD (1) C 3- H 8	3.3
BD (1) C 3- H 8	BD (1) C 1- H 11	4.2	BD (2) C 3- C 4	BD (1) C 1-S 2	19.5
BD (1) C 3- H 8	BD (1) C 1- H 13	4.1	BD (2) C 3- C 4	BD (1) C 1- H 11	2.2

Table S10: Observed rotational transitions for 2-MOT

J'	K'_a	K'_c	J''	K''_a	K''_c	Obs. (MHz)
2	0	2	1	0	1	6221.306
4	1	3	4	0	4	6273.006
3	0	3	2	1	2	6612.458
2	1	1	1	1	0	6756.081
11	4	7	10	5	6	7464.453
5	2	3	5	1	4	8101.863
5	1	4	5	0	5	8115.072
6	2	4	6	1	5	8349.112
3	2	1	3	1	2	8632.167
3	1	3	2	1	2	8657.679
2	1	2	1	0	1	8812.086
7	2	5	7	1	6	9086.999
2	2	0	2	1	1	9110.933
5	1	4	4	2	3	9133.339
3	0	3	2	0	2	9203.240
7	2	5	6	3	4	9361.375
3	2	2	2	2	1	9411.658
3	2	1	2	2	0	9620.082
3	1	2	2	1	1	10098.853
8	2	6	8	1	7	10388.320
6	1	5	6	0	6	10438.180
2	2	1	2	1	2	10502.654
3	1	3	2	0	2	11248.456
3	2	2	3	1	3	11256.650
4	1	4	3	1	3	11489.540

4	0	4	3	0	3	12052.299
4	2	3	4	1	4	12274.524
9	2	7	9	1	8	12282.604
9	3	6	9	2	7	12359.091
10	3	7	10	2	8	12368.853
4	2	3	3	2	2	12507.422
4	3	2	3	3	1	12644.970
4	3	1	3	3	0	12666.943
8	3	5	8	2	6	12810.163
4	2	2	3	2	1	13003.795
7	1	6	7	0	7	13141.835
5	0	5	4	1	4	13292.792
6	1	5	5	2	4	13304.109
4	1	3	3	1	2	13394.049
4	1	4	3	0	3	13534.746
7	3	4	7	2	5	13555.404
5	2	4	5	1	5	13557.820
8	2	6	7	3	5	14025.441
5	1	5	4	1	4	14284.804
6	3	3	6	2	4	14401.421
10	2	8	10	1	9	14730.350
5	0	5	4	0	4	14775.242
6	2	5	6	1	6	15099.552
5	3	2	5	2	3	15168.706
5	2	4	4	2	3	15568.105
4	3	1	4	2	2	15737.161
5	1	5	4	0	4	15767.263

5	4	2	4	4	1	15804.020
5	4	1	4	4	0	15805.705
2	2	1	1	1	0	15813.864
5	3	3	4	3	2	15833.195
5	3	2	4	3	1	15908.798
3	3	0	3	2	1	16074.021
8	1	7	8	0	8	16075.413
13	3	10	13	2	11	16137.579
14	4	10	14	3	11	16238.751
3	3	1	3	2	2	16331.895
2	2	0	1	1	1	16348.629
6	0	6	5	1	5	16423.755
13	4	9	13	3	10	16457.337
4	3	2	4	2	3	16469.441
5	2	3	4	2	2	16477.254
5	1	4	4	1	3	16617.294
5	3	3	5	2	4	16734.530
7	2	6	7	1	7	16882.269
6	1	6	5	1	5	17044.412
6	3	4	6	2	5	17172.099
6	0	6	5	0	5	17415.771
7	1	6	6	2	5	17446.485
11	2	9	11	1	10	17614.406
7	3	5	7	2	6	17821.906
13	6	8	12	5	7	76404.403
13	6	7	12	5	8	76623.804
9	8	1	8	7	2	77159.929

9	8	1	8	7	2	77159.929
9	8	2	8	7	1	77159.929
14	6	8	13	5	9	79761.584
10	8	2	9	7	3	80311.910
10	8	3	9	7	2	80311.910
15	6	10	14	5	9	81981.333
15	6	9	14	5	10	82958.143
13	7	7	12	6	6	83170.814
13	7	6	12	6	7	83181.293
11	8	3	10	7	4	83460.324
11	8	4	10	7	3	83460.324
9	9	0	8	8	1	83662.561
9	9	1	8	8	0	83662.561
14	7	8	13	6	7	86253.672
12	8	4	11	7	5	86603.285
12	8	5	11	7	4	86603.285
10	9	1	9	8	2	86816.697
10	9	2	9	8	1	86816.697
15	7	9	14	6	8	89298.855
17	6	11	16	5	12	89797.008
11	9	2	10	8	3	89969.334
11	9	3	10	8	2	89969.334
16	7	9	15	6	10	92444.705
12	9	3	11	8	4	93119.306
12	9	4	11	8	3	93119.306
10	10	0	9	9	1	93318.940
10	10	1	9	9	0	93318.940

17	7	11	16	6	10	95186.574
13	9	4	12	8	5	96265.258
13	9	5	12	8	4	96265.258
11	10	1	10	9	2	96473.168
11	10	2	10	9	1	96473.168
12	10	2	11	9	3	99626.216
12	10	3	11	9	2	99626.216

Table S11: Observed rotational transitions for 3-MOT

J'	K_a'	K_c'	J''	K_a''	K_c''	Obs. (MHz)
4	1	3	4	0	4	6143.279
1	1	1	0	0	0	6898.300
5	1	4	5	0	5	7248.550
6	1	5	5	2	4	7327.536
3	1	3	2	1	2	7924.748
3	0	3	2	0	2	8356.806
3	2	2	2	2	1	8433.840
3	2	1	2	2	0	8510.854
6	1	5	6	0	6	8683.118
3	1	2	2	1	1	8918.685
2	1	2	1	0	1	9378.031
7	1	6	7	0	7	10462.473
4	1	4	3	1	3	10545.322
7	2	5	7	1	6	10627.715
6	2	4	6	1	5	10722.312
8	2	6	8	1	7	10797.288
5	0	5	4	1	4	10854.848
5	2	3	5	1	4	11014.217
4	0	4	3	0	3	11055.333
7	1	6	6	2	5	11086.541
4	2	3	3	2	2	11230.033
4	3	2	3	3	1	11281.802
9	2	7	9	1	8	11285.211
4	3	1	3	3	0	11286.035
4	2	2	3	2	1	11419.785

4	2	2	4	1	3	11425.100
10	2	8	9	3	7	11848.658
4	1	3	3	1	2	11867.257
3	2	1	3	1	2	11872.576
10	2	8	10	1	9	12134.521
2	2	0	2	1	1	12280.399
8	1	7	8	0	8	12572.368
5	1	5	4	1	4	13149.896
2	2	1	2	1	2	13255.689
11	2	9	11	1	10	13376.497
5	0	5	4	0	4	13687.592
3	2	2	3	1	3	13764.772
4	1	4	3	0	3	13888.082
6	0	6	5	1	5	13957.358
5	2	4	4	2	3	14013.352
5	3	3	4	3	2	14115.797
5	3	2	4	3	1	14130.570
9	1	8	9	1	9	14304.233
5	2	3	4	2	2	14381.958
4	2	3	4	1	4	14449.487
5	1	4	4	1	3	14792.848
8	1	7	7	2	6	14897.137
9	1	8	9	0	9	14967.937
12	2	10	12	1	11	15025.368
5	2	4	5	1	5	15312.937
6	1	6	5	1	5	15736.799
5	1	5	4	0	4	15982.660

6	0	6	5	0	5	16252.426
14	3	11	14	2	12	16271.092
6	2	5	6	1	6	16356.843
12	3	9	12	2	10	16371.978
6	2	5	5	2	4	16780.701
15	3	12	15	2	13	16802.576
11	3	8	11	2	9	16875.793
6	5	2	5	5	1	16916.124
6	5	1	5	5	0	16916.124
6	4	3	5	4	2	16933.232
6	4	2	5	4	1	16933.999
6	3	4	5	3	3	16955.323
7	0	7	6	1	6	16980.900
6	3	3	5	3	2	16994.387
6	2	4	5	2	3	17395.120
10	3	7	10	2	8	17569.058
7	2	6	7	1	7	17580.421
6	1	5	5	1	4	17686.991

Table S12: Observed rotational transitions for 2-MST

J'	K'_a	K'_c	J''	K''_a	K''_c	Symmetry	Obs. (MHz)
6	2	5	6	1	5	blend	6458.734
8	2	6	7	3	4	blend	6551.530
3	1	3	2	1	2	blend	6902.881
5	2	4	5	1	4	blend	6948.543
3	0	3	2	0	2	blend	7137.753
4	4	0	5	3	2	E	7199.257
4	2	3	4	1	3	blend	7366.513
3	1	2	2	1	1	blend	7429.157
8	1	8	7	2	6	blend	7514.203
6	1	5	5	2	3	blend	7558.061
2	1	1	1	0	1	blend	7786.687
2	2	1	2	1	1	E	7964.286
2	2	1	2	1	1	A	7964.418
5	0	5	4	1	3	blend	8067.240
2	2	0	2	1	2	A	8499.356
2	2	0	2	1	2	E	8499.441
3	2	1	3	1	3	blend	8801.288
9	1	9	8	2	7	blend	9026.198
4	1	4	3	1	3	blend	9194.575
4	2	2	4	1	4	blend	9244.859
3	1	3	2	0	2	blend	9390.575
4	0	4	3	0	3	blend	9478.666
4	2	3	3	2	2	blend	9555.112
4	2	2	3	2	1	blend	9638.143
6	0	6	5	1	4	blend	9783.027

5	0	5	4	1	4	blend	9820.559
5	2	3	5	1	5	blend	9861.902
4	1	3	3	1	2	blend	9895.091
7	1	6	6	2	4	blend	10198.602
3	1	2	2	0	2	blend	10443.397
11	3	9	11	2	9	E	10678.725
11	3	9	11	2	9	A	10678.840
6	2	4	6	1	6	blend	10686.312
7	0	7	6	1	5	blend	11302.692
11	2	10	10	3	8	blend	11308.574
10	3	8	10	2	8	E	11421.499
10	3	8	10	2	8	A	11421.608
4	1	4	3	0	3	blend	11447.420
5	1	5	4	1	4	blend	11479.054
7	2	5	7	1	7	blend	11748.941
5	0	5	4	0	4	blend	11789.306
5	2	4	4	2	3	blend	11933.326
5	4	2	4	4	1	blend	11970.629
5	3	3	4	3	2	A	11978.366
5	3	3	4	3	2	E	11978.841
5	3	2	4	3	1	E	11983.145
5	3	2	4	3	1	A	11983.644
9	3	7	9	2	7	E	12062.450
9	3	7	9	2	7	A	12062.550
5	2	3	4	2	2	blend	12096.105
5	1	4	4	1	3	blend	12351.305
10	2	8	9	3	6	A	12360.157

10	2	8	9	3	6	E	12360.249
6	0	6	5	1	5	blend	12408.609
8	3	6	8	2	6	E	12590.666
8	3	6	8	2	6	A	12590.763
8	0	8	7	1	6	blend	12618.736
2	2	1	1	1	0	E	12920.672
2	2	0	1	1	0	A	12929.199
2	2	0	1	1	0	E	12929.273
7	3	5	7	2	5	E	13001.757
7	3	5	7	2	5	A	13001.842
8	2	6	8	1	8	A	13072.513
8	2	6	8	1	8	E	13072.614
2	2	1	1	1	1	E	13096.182
2	2	1	1	1	1	A	13096.296
4	1	3	3	0	3	blend	13200.745
6	3	4	6	2	4	E	13299.706
6	3	4	6	2	4	A	13299.869
5	1	5	4	0	4	blend	13447.791
5	3	3	5	2	3	E	13497.473
5	3	3	5	2	3	A	13497.766
5	3	2	5	2	3	E	13505.030
4	3	2	4	2	2	E	13614.731
4	3	2	4	2	2	A	13615.518
4	3	1	4	2	2	E	13617.994
3	3	1	3	2	1	E	13674.660
3	3	1	3	2	1	A	13675.963
3	3	0	3	2	1	E	13677.403

3	3	1	3	2	2	E	13716.657
3	3	0	3	2	2	A	13718.172
3	3	0	3	2	2	E	13719.404
9	0	9	8	1	7	E	13736.975
9	0	9	8	1	7	A	13737.124
4	3	2	4	2	3	E	13739.749
4	3	1	4	2	3	A	13742.291
4	3	1	4	2	3	E	13743.010
6	1	6	5	1	5	blend	13755.350
5	3	3	5	2	4	E	13785.265
5	3	3	5	2	4	A	13785.526
5	3	2	5	2	4	A	13792.593
5	3	2	5	2	4	E	13792.825
6	3	3	6	2	5	A	13883.880
6	3	3	6	2	5	E	13883.948
7	3	4	7	2	6	blend	14035.253
6	0	6	5	0	5	blend	14067.097
8	3	5	8	2	7	blend	14270.482
6	2	5	5	2	4	blend	14304.565
6	3	4	5	3	3	A	14381.863
6	3	4	5	3	3	E	14382.017
6	3	3	5	3	2	E	14395.687
6	3	3	5	3	2	A	14395.870
6	2	4	5	2	3	blend	14579.774
6	2	4	5	2	3	A	14579.774
9	3	6	9	2	8	blend	14618.416
9	2	7	9	1	9	blend	14669.229

6	1	5	5	1	4	blend	14794.388
7	0	7	6	1	6	blend	14967.275
13	2	12	12	3	10	blend	15036.026
10	3	7	10	2	9	blend	15112.467
9	1	8	8	2	6	blend	15133.784
3	2	2	2	1	1	E	15135.630
3	2	2	2	1	1	A	15135.696
3	2	1	2	1	1	blend	15177.656
11	2	9	10	3	7	blend	15320.467
6	1	6	5	0	5	blend	15413.832
3	2	2	2	1	2	blend	15662.197
3	2	1	2	1	2	blend	15704.164
7	1	7	6	1	6	blend	16023.007
5	1	4	4	0	4	blend	16073.380
7	0	7	6	0	6	blend	16314.021
7	2	6	6	2	5	blend	16667.492
7	6	1	6	6	0	blend	16755.780
7	6	2	6	6	1	blend	16755.780
7	5	3	6	5	2	blend	16762.283
7	5	2	6	5	1	blend	16762.283
7	4	4	6	4	3	A	16774.161
7	4	4	6	4	3	E	16774.438
7	4	3	6	4	2	E	16774.575
7	4	3	6	4	2	A	16774.907
7	3	5	6	3	4	blend	16787.639
7	3	4	6	3	3	E	16818.776
7	3	4	6	3	3	A	16818.867

14	4	11	14	3	11	E	16960.218
14	4	11	14	3	11	A	16960.363
7	2	5	6	2	4	blend	17085.613
7	1	6	6	1	5	blend	17220.313
4	2	3	3	1	2	blend	17261.629
7	1	7	6	0	6	blend	17369.773
4	2	2	3	1	2	blend	17386.617

Table S13: Observed rotational transitions for 3-MST

J'	K_a'	K_c'	J''	K_a''	K_c''	Obs. (MHz)
3	0	3	2	0	2	6203.363
6	1	5	6	0	6	6475.365
3	1	2	2	1	1	6571.320
7	1	6	6	2	5	6585.811
2	1	2	1	0	1	7447.463
5	0	5	4	1	4	7575.804
7	1	6	7	0	7	7614.412
4	1	4	3	1	3	7861.105
4	0	4	3	0	3	8224.419
4	2	3	3	2	2	8317.639
4	1	3	3	1	2	8749.082
8	1	7	8	0	8	8984.380
7	2	5	7	1	6	9105.365
9	2	7	9	1	8	9183.374
3	1	3	2	0	2	9199.194
6	2	4	6	1	5	9296.571
8	1	7	7	2	6	9393.613
10	2	8	10	1	9	9523.844
10	2	8	10	1	9	9523.851
5	2	3	5	1	4	9582.798
5	1	5	4	1	4	9809.107
4	2	2	4	1	3	9915.003
6	0	6	5	1	5	9918.205
5	0	5	4	0	4	10208.311
3	2	1	3	1	2	10245.241

5	2	4	4	2	3	10384.246
5	3	3	4	3	2	10439.216
5	3	2	4	3	1	10445.433
2	2	0	2	1	1	10531.491
9	1	8	9	0	9	10574.658
5	2	3	4	2	2	10582.901
4	1	4	3	0	3	10856.918
5	1	4	4	1	3	10915.087
12	2	10	12	1	11	10939.357
2	2	1	2	1	2	11188.557
3	2	2	3	1	3	11528.486
6	1	6	5	1	5	11747.037
4	2	3	4	1	4	11985.023
13	2	11	13	1	12	12047.156
6	0	6	5	0	5	12151.498
7	0	7	6	1	6	12226.928
9	1	8	8	2	7	12236.220
10	1	9	10	0	10	12358.393
5	1	5	4	0	4	12441.604
6	2	5	5	2	4	12442.361
6	3	3	5	3	2	12553.233
5	2	4	5	1	5	12560.189
6	2	4	5	2	3	12779.020
6	1	5	5	1	4	13065.255
6	2	5	6	1	6	13255.520
7	1	7	6	1	6	13674.312
6	1	6	5	0	5	13980.312

7	0	7	6	0	6	14055.734
7	2	6	7	1	7	14071.577
8	0	8	7	1	7	14480.809
2	2	1	1	1	0	14906.491
7	2	5	6	2	4	15003.547
8	2	7	8	1	8	15007.512
2	2	0	1	1	1	15139.182
7	1	6	6	1	5	15194.785
7	1	7	6	0	6	15503.121
8	1	8	7	1	7	15590.839
10	3	7	10	2	8	15734.604
9	2	8	9	1	9	16060.677
9	3	6	9	2	7	16319.905
8	2	7	7	2	6	16526.767
9	0	9	8	1	8	16669.092
3	2	2	2	1	1	16765.464
8	3	5	7	3	4	16811.789
8	3	5	8	2	6	16846.559
8	1	8	7	0	7	17038.215
10	2	9	10	1	10	17226.382
8	2	6	7	2	5	17247.436
7	3	4	7	2	5	17282.193
8	1	7	7	1	6	17298.159
3	2	1	2	1	2	17483.864
9	1	9	8	1	8	17497.093
6	3	3	6	2	4	17611.838
9	0	9	8	0	8	17779.111
